# Creation of Text Vignettes Based on Patient-Reported Data to Facilitate a Better Understanding of the Patient Perspective: Design Study

**DOI:** 10.2196/58077

**Published:** 2025-02-05

**Authors:** Sofia Charlotte Kelly, Emilie Kauffeldt Wegener, Lars Kayser

**Affiliations:** 1 Department of Public Health University of Copenhagen Copenhagen Denmark

**Keywords:** patient-reported outcome, text vignette, data representation, Readiness and Enablement Index for Health Technology, understanding, health care system, data analysis, clinical training, clinician, health professional

## Abstract

**Background:**

Patient-reported outcome (PRO) data refer to information systematically reported by patients, or on behalf of patients, without the influence of health care professionals. It is a focal point of the health care system’s ambition toward becoming more involving and personalized. It is recognized that PROs provide valuable data. However, despite this recognition, there are challenges related to both patients’ and clinicians’ accurate interpretations of the quantitative data. To overcome these challenges, this study explores text vignettes as a representation of PROs.

**Objective:**

This study aimed to develop data-informed text vignettes based on data from the Readiness and Enablement Index for Health Technology (READHY) instrument as another way of representing PRO data and to examine how these are perceived as understandable and relevant for both patients and clinicians.

**Methods:**

The text vignettes were created from participant responses to the READHY instrument, which encompasses health literacy, health education, and eHealth literacy. The text vignettes were created from 13 individual text strings, each corresponding to a scale in the READHY instrument. This study consisted of 3 sequential parts. In part 1, individuals with chronic obstructive pulmonary disease completed the READHY instrument, providing data to be used to create vignettes based on cluster profiles from the READHY instrument. Part 2 focused on the development of scale-based strings representing all READHY dimensions, which were evaluated through iterative cognitive interviews. In part 3, clinicians and patients assessed the understanding and relevance of the text vignettes.

**Results:**

Clinicians and patients both understood and related to the text vignettes. Patients viewed the text vignettes as an accurate reflection of their PRO responses, and clinicians perceived the text vignettes as aligned with their understanding of patients’ experiences.

**Conclusions:**

Text vignettes can be developed using PRO instruments, with individual scales as input strings. This provides an opportunity to present numeric values in a text format that is understandable and recognizable to most patients and clinicians. Challenges with the vignette’s language and layout require customization and clinician training to ensure meaningful interpretation. Findings also support the need to expand the study and enhance clinical relevance with alternative or contextually relevant text vignettes.

## Introduction

### Background

Patient-reported outcome (PRO) data represent patients’ self-assessed health status in terms of physical and mental health and do not involve health professionals [1]. PRO data can be used as a screening tool before outpatient visits as a supplement to in-person or virtual consultations, serving as a stratification tool to reduce the number of visits needed. PRO data can also be used during consultations to screen for symptoms or serve as a dialogue and decision tool. The use of PRO data during consultations may increase the patient’s self-awareness and health literacy, facilitate joint decision-making, enable self-management, and lead to better health outcomes [1,2].

### Health Literacy

Competence in health literacy and digital knowledge is fundamental for an accurate understanding of PRO data as well as knowledge about health behavior and health practices and how patients understand and interpret health information [3]. Challenges arise when data accessibility does not consider differences in health literacy. While aggregate-level understanding may tolerate some variations, individual patient pathways require a common understanding to capture nuances [4,5]. Therefore, it is crucial to account for patients’ health literacy and cognition in the clinical use of PROs.

### Interpretation of PRO Data

When incorporating PROs into individual patient pathways, it becomes crucial to ensure that patients fully understand the questions, fostering a shared understanding. In working with PRO data, an assumption is often made that people have the same needs, preferences, perceptions, and experiences and will attach similar importance to items in the questionnaire, thus leaving no room to capture different additions of value or uncover nuances [4]. The quantitative part of PRO data operates within a set of assumptions about the world, which states that the standardized numerical score is the objective representation of the respondents’ health status and health-related quality of life, independent of an individual’s unique life story [4].

For both clinicians and patients, it may be difficult to understand the meaning of PRO data when they are presented as values. Patients often prefer visual representations, such as bar charts and line charts, which prove useful for quick comprehension and comparisons over time [6]. However, the accuracy of interpretation is closely tied to the individuals’ prior knowledge of these graphical representations [6].

Graphical presentations may be a challenge. Grossman et al [7] highlighted that individuals lacking prior knowledge of bar charts struggled to interpret them accurately. Furthermore, health literacy plays a crucial role in correct interpretation, with studies suggesting that patients with lower health literacy may find it challenging to grasp the longitudinal nature of PRO data [8]. There is a notable correlation between educational level and accurate interpretation, along with age-related difficulties in correctly interpreting graphic representations of PRO data [6,9]. Patients generally, regardless of educational level, prefer less-complex presentations of PRO data, but a correlation has been demonstrated between age and accurate interpretation, where patients aged >65 years had substantially more difficulty interpreting graphic representations of PRO data correctly [6,9]. In Denmark, 1 in 5 individuals is aged >65 years [10], which means that a relatively large proportion of all individuals will potentially have difficulty interpreting PRO data.

The use of traffic light colors, that is, green, yellow, and red, in representing PRO data adds another layer of complexity. While this method increases accuracy in interpretation across various graphical representations [6,11], it is not foolproof and 93% of patients in a study population were able to correctly interpret the meaning of traffic light colors [6]. Despite this visual aid, there remains a need for additional descriptive explanations to enhance understanding [9].

Patients’ and health care professionals’ perceived understanding of graphically presented PRO data often exceeds their actual comprehension [9]. In addition, studies indicate that there may be a discrepancy between patients’ preferred form of visualization and the form of visualization they can interpret with the highest accuracy [9].

Patients may also view the use of PRO data in treatment as impersonal and nonbeneficial for the relationship between themselves and their clinicians because they experience that the focus shifts from their own perspective to quantitative goals [12]. This perspective highlights the significance of maintaining a patient-centered approach.

### A Common Understanding of PROs

When patients and clinicians collaborate on data interpretation, potential errors may arise due to questionnaire misinterpretation. This divergence in understanding creates a need for a common language or understanding of the representations of colors, figures, or text. A challenge is that patients and clinicians possess distinct cognitive frameworks influencing their actions and comprehension [13]. Data interpretation involves understanding available data, extracting information, and supplementing it for comprehensive comprehension [13]. Clinicians are shaped by a positivist background and may differ from patients in cognitive approaches, impacting data interpretation. When PRO data are translated into quantitative targets, it may be unclear what data have been made available and what information can be extracted. Patients provide a wide range of data, but data visualization often only includes part of these data.

Despite psychometric validation, there is a need to address discordance in individual interpretations of PRO data. Discordance reasons include word interpretation mismatches, evolving patient circumstances, and differing expectations [14]. Awareness of these differences is crucial in interpreting PRO data [14]. PRO tools vary in complexity, leading to further potential errors if patients lack an accurate understanding. A study found that only 11% of 59 PRO questionnaires are readable by the average UK adult [15], emphasizing the need for a patient-clinician dialogue to enhance understanding and decision-making [5]. Numerical scores derived from PROs can represent a person’s health status or health-related quality of life to a limited extent because some of the narrative is lost along the way. In the representation of PRO data, there is a need to be able to give meaning to individual experiences [4]. Positivism argues that knowledge should be based on the objective world via empirically verified causal explanations based on “positive” facts and not on abstract inferences; however, according to Meadow [4], we cannot understand the patient if we do not also try to understand subjective and emotional meaning.

### PROs as Text Vignettes

One approach to a more understandable and personalized representation of data about a group of patients or individuals is vignettes created based on a combination of clustered data, which are enriched with sociodemographic characteristics and interviews with individuals representing these clusters. An example of this is the Optimizing Health Literacy and Access (OPHELIA) process, where issues related to health care access and engagement are identified and addressed [16,17]. This way of creating data-informed text vignettes is a powerful tool to provide clinicians and policy makers with insights into a more detailed description of archetypes of patients [18]. In this way, text vignettes play a pivotal role in providing a nuanced understanding of the perceived status of various subgroups of patients in a specific context or area. The primary aim is to bring to life the “person behind the numbers,” referring to PRO scores, addressing the challenge of visualizing the individuals behind the statistical data. To the best of our knowledge, this has not yet been done at the level of individuals by creating person-specific text vignettes.

In this study, inspired by the OPHELIA process, we will explore whether text vignettes can be developed from more structured text inputs and how this work can be advanced to create text vignettes that can be used at the individual level, rather than at the group level, to present personalized texts that reflect each patient’s perception of their condition. The study uses the Readiness and Enablement Index for Health Technology (READHY) [19], which covers 3 key themes—self-management, social capital, and digital health literacy. This instrument was selected because, as its developers, we have extensive experience with its performance, and its design makes it well suited as a PRO tool for telehealth services. The study demonstrates the process of converting psychometric instrument scales into data-informed text vignettes, highlighting the challenges and implications to consider for ensuring understanding and relevance for both patients and clinicians.

### Objectives

This study aimed to develop data-informed text vignettes based on data from the READHY instrument as another way of representing PRO data and to examine how these are perceived as understandable and relevant for both patients and clinicians.

### Part 1 Introduction: Generating Vignettes Based on READHY Clusters Enriched With Sociodemographic Data

This part is a proof of concept, where cluster-based profiles were created to help develop representatives of characteristics of a patient population divided into subgroups. This enabled us to work with materials based on real-life data varying with respect to the 13 scale scores. This has provided insights into how dimension names can be phrased for text vignettes without a systematic approach, while also helping us establish a shared understanding of the OPHELIA [16] principles. These principles were operationalized in a data-informed manner, bridging the previously reported qualitative approach with a more quantitative-based methodology.

### Part 2 Introduction: Creation of Text Strings and Testing of Meaning in Vignettes

In part 2, scale-based text strings describing the different dimensions measured in READHY were developed through a series of iterations. These were then combined into comprehensive text vignettes profiling the participants. For this process, the upper threshold was adjusted from 2.50 to 2.70 based on data from the study by Kayser et al [20]. The vignettes were continuously refined through cognitive interviews to evaluate and improve the participants’ understanding and ability to comprehend the content.

### Part 3 Introduction: Applicability of Text Vignettes in a Clinical Setting

The text strings iteratively created and validated in part 2 served as the foundation for the work in part 3. In part 3, the focus shifted to testing the applicability of individual text vignettes within a clinical setting. The emphasis was on how meaningful and relevant these text vignettes were in a setting involving both patients and clinicians within a telehealth service, where the READHY instrument was suitable as a PRO instrument. The clinical setting included here was the same as the one used to gather data for part 1.

## Methods

### Overview

**Figure 1:**
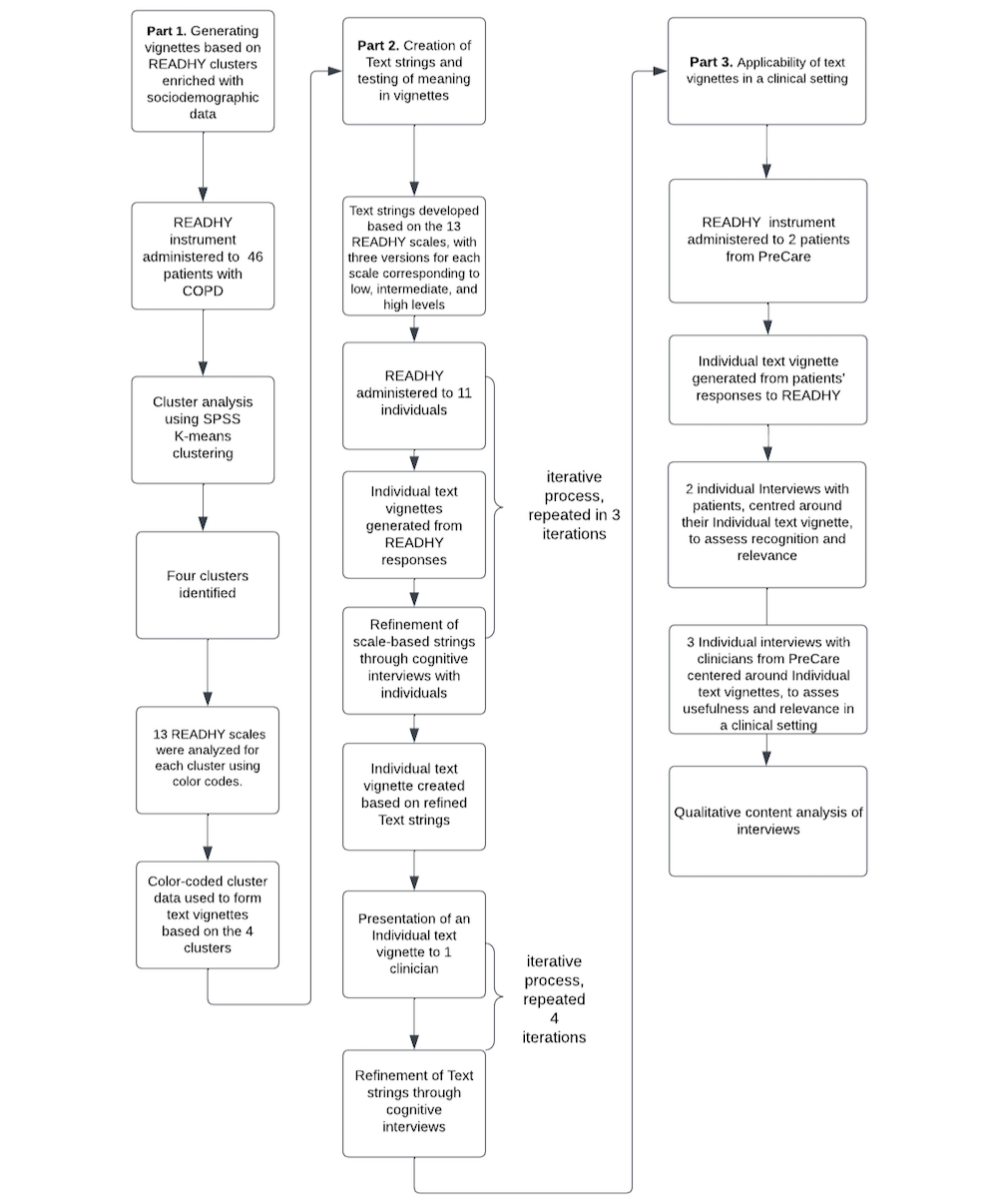
Flowchart: the 3-part process for examining the use of text vignettes. COPD: chronic obstructive pulmonary disease; READHY: Readiness and Enablement Index for Health Technology.

The study comprises 3 parts, each building upon the previous one. In part 1, which was inspired by the OPHELIA process [16], the READHY instrument was administered to individuals living with chronic obstructive pulmonary disease (COPD). Data gathered from this group of participants served as the foundation for generating 4 clusters, which were characterized by high, intermediate, and low levels of the 13 READHY scales, which together with another Health Literacy Questionnaire (HLQ) scale, “actively managing my health and sociodemographics”, were used to develop a proof of concept of how data-informed text vignettes could be used to illustrate the quantitative data qualitatively to better understand profiles of user groups. The text vignettes in part 2 were crafted as narratives, emphasizing the group’s high or low scores. This approach created profiles that highlighted their resources and barriers. In part 2, we expanded on the idea by constructing scale-specific strings for each of the 3 levels for each scale in READHY. The text strings were created to constitute a list of variables, which in a later process could be combined based on an algorithm to form the text vignettes. This computer-based combination of the text strings is beyond the scope of this study that only addresses the development of our concept up to a technology readiness level of 3 [21]. Text vignettes are consequently created by manually combining the text strings. In this process, the text strings were evaluated for clarity and understanding through multiple iterations of cognitive interviews with a diverse set of participants. In part 3, the refined scale-based strings were combined manually, informed by the 2 patients’ READHY scores, into individual text vignettes, which were presented to clinicians and patients for assessment of their understanding, relevance, and recognizability (Figure 1).

### Setting and Population

The setting for the reported activities was in part 1 and part 3 of a telehealth service, PreCare [22], which provides 24-7 access to a response and coordination center manned with registered nurses and backed up by an e-doctor. PreCare is organized based on the Epital care model [22,23]. The participants in part 2 were recruited broadly among individuals in the eastern part of Denmark and clinicians not currently working in telehealth.

### Participants

#### Part 1

The participants were 46 adults living with COPD, with a mean age of 74.4 (SD 6.8; range 59-91) years. They were patients recruited by convenience sampling from the European Commission funded project Smart Inclusive Living Environments, were enrolled in the PreCare project in Region Zealand, and were diagnosed with different severity levels of COPD [24]. The inclusion criteria were a COPD diagnosis and being active in the PreCare project. The patients should be able to understand and communicate in Danish.

#### Part 2

##### Individuals

A total of 11 individuals were recruited using a snowball technique in the Capital Region of Denmark and Region Zealand. Initially, 3 members of interest were identified and approached within their local areas. These individuals were then asked to refer others in their community who might be willing to participate. With their consent, we received the necessary contact information and subsequently reached out to the referred participants by phone to arrange their involvement in the study. The inclusion criteria for individuals’ participation was that they had to be aged >18 years, able to understand Danish, capable of giving informed consent, and able to complete the READHY instrument, with assistance if needed (ie, the ability to read and write independently was not a criterion). The first 3 recruited individuals resided in the metropolitan area, had medium-length higher education (International Standard Classification of Education level 6-7) [25], and had an average age of 53 (SD 12.36) years. This group was expanded to include 8 participants, aged between 36 and 86 years, from the capital region, Lolland, and Jutland with educational backgrounds ranging from International Standard Classification of Education level 4 to 6 [25] to attain a more diverse and comprehensive perspective.

##### Clinicians

A total of 4 clinicians were also recruited using a snowballing technique. Initially, 3 clinicians were approached, but not all expressed interest in participating. These contacts referred to others, and with their consent, we obtained their contact information and contacted them. The clinicians who participated in the interviews were 1 registered nurse, 2 physicians, and 1 professional with a master’s and PhD degree in biomedicine. Of the 4 physicians, 2 (50%) had prior knowledge of READHY as active researchers using the instrument. All physicians were involved in professional settings related to digital health in patient care, and they were all familiar with PROs.

#### Part 3

A total of 3 registered nurses and 2 patients from the PreCare clinic were interviewed about the understanding, recognizability, and relevance of individual text vignettes in a clinical setting.

The 2 patients, a woman aged 75 years and a man aged 80 years, lived in the region where the PreCare project is based. They were selected by PreCare staff based on the following inclusion criteria: a COPD diagnosis, active participation in PreCare, the ability to understand and communicate in Danish, clinic enrollment at the time of data collection, and prior consent through PreCare as well as informed consent specifically for this study. A total of 3 (75%) of the 4 nurses in PreCare were available for interviews.

### Application of the READHY Instrument

READHY is a validated PRO instrument that combines several aspects of PROs to illuminate experienced support, digital health literacy, and coping with everyday life in relation to one’s health [19]. The tool focuses on aspects of knowledge and skills related to health; self-care, disease, and health-related mindset; experiences with health technology and the individual understanding of these; as well as the extent to which users feel supported by relatives and health care professionals [19].

READHY has been used to identify different health technology readiness profiles within different patient groups, such as patients living with type 2 diabetes and patients with cancer [26,27].

READHY consists of 65 items comprising 13 dimensions or scales. The 13 scales are selected from 3 PRO instruments. Four scales are from the Health Education Impact Questionnaire (heiQ), which evaluates the effect of health education interventions on self-care [28]. These address aspects of self-management with relevance for a digital health context, such as insights and attitudes. Two scales from the HLQ, which assesses health literacy, including support from relatives, peers, and health professionals [29], and all 7 scales from the eHealth Literacy Questionnaire (eHLQ), which address patients’ digital health literacy, were included [30]. The eHLQ informs about knowledge, skills, motivation, perceptions, and experiences in relation to digital health. The 13 dimensions of READHY, which correspond to its 13 scales, are presented in Textbox 1.

**The 13 dimensions of the Readiness and Enablement Index for Health Technology framework.**

- Health Education Impact Questionnaire (heiQ) 3: self-monitoring and insight
- heiQ4: constructive attitudes and approaches
- heiQ5: skill and technique acquisition
- heiQ8: emotional distress
- Health Literacy Questionnaire (HLQ) 1: feel understood and supported by health care providers
- HLQ4: social support for health
- eHealth Literacy Questionnaire (eHLQ) 1: using technology to process health information
- eHLQ2: understanding of health concepts and language
- eHLQ3: ability to actively engage with digital services
- eHLQ4: feel safe and in control
- eHLQ5: motivated to engage with digital services
- eHLQ6: access to digital services that work
- eHLQ7: digital services that suit individual needs

The READHY instrument uses a 4-point response scale for all items in the instrument [19], where 1 represents strongly disagree, 2 represents disagree, 3 represents agree, and 4 represents strongly agree. The results are 13 scale scores. For the scale heiQ8 (emotional distress), which includes 6 items, the scale is reversed by subtracting the scored value from 5. This adjustment simplifies interpretation, as higher scores now correspond to less impact.

### Ethical Considerations

Written informed consent was obtained from all participants after providing information in accordance with the Helsinki II Declaration, including their right to opt out. Participants did not receive any salary or reimbursement of costs. Health science questionnaire surveys and interview studies that do not involve human biological material (section 14(2) of the Danish Act on Committees) do not require reporting or approval from the Danish National Center for Ethics [31].

### Part 1 Methods

#### Data Sampling

The READHY instrument was handed out and collected with a combination of mail using prestamped envelopes by EKW and a researcher in PreCare in May 2021. A total number of 120 surveys including the READHY instrument were distributed.

#### Cluster Analysis

In managing the READHY data from the 46 patients, clusters were created using the K-means clustering function in IBM’s software package SPSS Statistics (version 24) [32]. Three models were created with 3, 4, and 5 clusters. On the basis of an evaluation of which cluster model would be most meaningful from a clinical perspective, a 4-cluster model was selected. The final cluster constituted 41 valid cases and 5 missing cases out of the 46 responses. The number of cases in each cluster was as follows: 16 in cluster 1, 5 in cluster 2, 11 in cluster 3, and 9 in cluster 4.

The cluster data were then used to form the group-based text vignettes. For each cluster, the 13 scales were initially labeled with a color indicating whether the level was low, intermediate, or high. We used arbitrary thresholds informed by a regional survey, Sundhedsprofilen 2021 [33], which is the region where the PreCare project is based. All values <2.00 were assigned red, all values between 2.01 and 2.50 were assigned yellow, and values >2.50 were given green. This helped us create text strings that were specific for each of the colors indicating high or low average scores for each scale and assigning them to the cluster text vignette. The text strings were created from the dimension name and informed by the items in the scale. Additional data from an additional HLQ scale, “actively managing my health,” and sociodemographic variables, such as educational level, experience living with, and number of chronic conditions, were added to the profiles to enrich them and create personas that were recognizable to the clinicians.

### Part 2 Methods

#### Overview

The overall process for constructing the text vignettes involved several steps. First, text strings were developed based on the 13 READHY scales, with 3 versions for each scale corresponding to low, intermediate, and high levels. Next, participants completed the instrument. The average score for each scale was then manually calculated from their responses, and this average score was matched with the corresponding text string for that scale score. Finally, the 13 text strings were combined into an individual text vignette. The text strings were combined in a coherent order, prioritizing readability and clarity, rather than strictly following the numerical order of the scales. In part 2, the focus was only on the READHY scales and text vignettes; therefore, part 2 did not include sociodemographic information, as these are anticipated to be retrieved from other data sources such as electronic health records as they are specific at the individual level.

#### Constructing Data-Informed Text Vignettes

The text vignettes were created by combining 13 text strings into a cohesive PRO profile. Each sentence in the text vignette corresponds to a specific scale in READHY, describing the knowledge, skills, and attitudes that the scale items aim to capture.

Initially, 39 text strings were constructed—3 for each of the 13 READHY scales, representing different levels of knowledge, skills, or attitudes within each scale. These initial text strings were closely aligned with the scale names, as the scale names summarized the experiences the items were designed to encompass. For example, the scale “self-monitoring and insight” (heiQ3) assesses an individual’s ability to monitor their health and their insight into their health [19]. An initial sentence for this scale might read as follows: “Has good insight into and control over his/her health and has a constructive attitude and approach to it.”

The levels are determined based on the average score for each scale, with 3 categories: level 1 indicates problematic knowledge, skills, and attitudes; level 2 corresponds to limited or insufficient knowledge, skills, and attitudes; and level 3 signifies adequate knowledge, skills, and attitudes. To be classified as level 3, the average score must be >2.7, indicating agreement or strong agreement with the statement (Table 1). The cutoffs are estimates defined by the authors and have not been validated by other parties. However, they have been used and found relevant in other studies using HLQ and eHLQ scores [20,33].

**Table 1:** Breakdown of knowledge, skills, and attitude into levels based on average scores.

Average scale score^a^	Level of knowledge, skills, and attitudes
<2.00	Level 1
2 to 2.7	Level 2
>2.7	Level 3

^a^The cutoffs are author-defined estimates, unvalidated by others but found relevant in studies using the Health Literacy Questionnaire and eHealth Literacy Questionnaire [20,33].

These 39 text strings are organized in Table 2 with the 13 scales as rows and the 3 levels as columns. To construct a text vignette, the appropriate text strings were selected from the table based on the participant’s average scale score for each scale. The 13 selected text strings were then manually combined into a coherent text vignette. The initial text strings were revised throughout part 2 of the study in an iterative process using cognitive interviews for accuracy and relevance.

**Table 2:** Refined text strings based on scores.

Scale	Scores <2.00	Scores between 2.00 and 2.70	Scores >2.70
heiQ3^a^: self-monitoring and insight	He/she experiences having insufficient insight into his/her state of health, with insufficient knowledge of how to deal with health problems when they arise.	He/she experiences having limited insight into his/her state of health, with limited knowledge of how to deal with health problems when they arise.	He/she experiences having good insight into his/her state of health, with sufficient knowledge of how to deal with health problems when they arise.
heiQ4: constructive attitudes and approaches	He/she finds that his/her health problems limit him/her from enjoying life.	He/she finds that his/her health problems limit him/her from enjoying life to some extent.	He/she does not find that his/her health problems limit him/her from enjoying life.
heiQ5: skill and technique acquisition	He/she experiences having insufficient skills and techniques to deal with health problems when they arise.	He/she experiences having limited skills and techniques to deal with health problems as they arise.	He/she experiences having sufficient skills and techniques to deal with health problems as they arise.
heiQ8: emotional distress	He/she generally experiences a high degree of emotional distress due to his/her health.	He/she generally experiences a bit of emotional distress due to his/her health.	He/she generally experiences no emotional distress due to his/her health.
HLQ4^b^: social support for health	He/she does not feel understood or supported by his/her social network.	He/she feels only partially understood and supported by his/her social network.	He/she feels adequately understood and supported by his/her social network.
HLQ1: feel understood and supported by health care providers	He/she experiences a lack of understanding and support from the health care professionals he/she has contact with and access to.	He/she experiences limited understanding and support from the health care professionals he/she has contact with and access to.	He/she experiences adequate understanding and support from the health care professionals she/he has contact with and access to.
eHLQ2^c^: understanding of health concepts and language	He/she experiences having trouble understanding information about health and illness and may experience insufficient knowledge to have conversations with others about this.	He/she may experience problems understanding information about health and illness and may experience having limited knowledge to have conversations with others about this.	He/she does not experience having problems understanding information about health and illness and has enough knowledge to have conversations with others about this.
eHLQ1: using technology to process health information	He/she finds that he/she has have insufficient knowledge about how he/she can use technology to take care of his/her health.	He/she finds that he/she has limited knowledge about how he/she can use technology to take care of his/her health.	He/she has sufficient knowledge about how he/she can use technology to take care of his/her health.
eHLQ3: ability to actively engage with digital services	He/she has insufficient knowledge of how to use technology to navigate health care systems.	He/she has limited knowledge of how to use technology to navigate health systems.	He/she has good knowledge of how to use technology and can navigate health systems easily.
eHLQ6: access to digital services that work	He/she experiences insufficient access to digital health systems that work and can be accessed by himself/herself and others who need it.	He/she experiences having limited access to digital health systems that work and can be accessed by himself/herself and others who need it.	He/she experiences having sufficient access to digital health systems that work and can be accessed by himself/herself and others who need it.
eHLQ7: digital services that suit individual needs	He/she believes that digital services are insufficiently able to adapt to his/her needs.	He/she believes that digital services can adapt to his/her needs to a limited extent.	He/she believes that digital services can adequately adapt to his/her needs.
eHLQ5: motivated to engage with digital services	He/she finds that technology is insufficiently helpful and helps him/her keep up with and take care of his/her health.	He/she finds that technology is helpful to a limited extent and helps him/her keep up with and take care of his/her health.	He/she finds that technology is sufficiently useful and helps them keep up with and take care of his/her health.
eHLQ4: feel safe and in control	He/she feels uneasy about how his/her health data are used by others.	He/she feels partly uncomfortable with how his/her health data are used by others.	He/she feels comfortable with how his/her health data are used by others.

^a^heiQ: Health Education Impact Questionnaire.

^b^HLQ: Health Literacy Questionnaire.

^c^eHLQ: eHealth Literacy Questionnaire.

#### Data Sampling and Analysis

Data collection involved conducting cognitive interviews with each participant to validate the individual text vignettes developed from the individuals’ READHY responses. These interviews allowed for iterative feedback and refinement of the text string to ensure their coherence and relevance. The analysis focused on identifying and addressing any issues in understanding and meaningfulness of the text strings and vignettes.

#### Iterative Validation of Data-Informed Text Vignettes

The text strings were validated through cognitive interviews with individuals and clinicians in an iterative process. Cognitive interviews, often used for validating questionnaires, involve participants “thinking out loud” by verbally reporting their mental activity while engaging with an item in a questionnaire [34,35]. These interviews are conducted using a semistructured approach, with in-depth sessions where participants read a text vignette aloud and provide feedback by sharing their thoughts and insights throughout the process.

The interviews were conducted in 2 stages: first with individuals and then with clinicians. Before each individual’s interview, they completed READHY, which was then used to create individual text vignettes. Each individual was presented with a full vignette, created based on their previous responses, to gather feedback on both individual text strings and the vignette as a coherent whole, evaluating its clarity and personal relevance.

After each interview, the data were analyzed individually to identify issues in the understanding and meaningfulness of the text vignettes. These individual findings were then aggregated across interviews to identify recurring problems, leading to adjustments of the text vignettes.

The iterative process included multiple stages of feedback incorporation. The first iteration considered feedback from 3 participants, with subsequent iterations incorporating feedback from additional participants—3 in the second and third iterations and 2 in the fourth.

Identified problems were categorized informally into 4 groups: scale-specific wording changes, need for further specification, issues with text vignette setup, and difficulties in answering the instrument [34].

Once all individual interviews were completed and the text vignettes and algorithm were adjusted, clinicians were introduced to the revised vignettes. The assignment of vignettes to clinicians was random, as they had no prior knowledge of or relationship with the individuals. Clinicians provided feedback on these vignettes, which led to further refinements. Each revised version was then presented to subsequent clinicians for additional input, ensuring that the final text was coherent and well understood.

### Part 3 Methods

#### Data Sampling

First, 2 patients completed the instrument. From their responses, 2 individual text vignettes were constructed using the same method as applied in part 2. The average scale scores were calculated for each scale, and the scale score was then matched with the final adjusted corresponding sentence (Table 2). Afterward, the 13 text strings were combined to create an individual text vignette. These text vignettes were then presented to the 3 clinicians in 3 individual semistructured interviews designed to explore the text vignettes within an actual clinical environment and gain the clinicians’ perspective. Each clinician was presented with 1 text vignette. Because all clinicians were somewhat familiar with booth patients, the specific vignette used in the interviews was randomly chosen based on which vignette was completed at the time of the interview.

Patients were also interviewed individually to assess their recognition of themselves in their text vignettes and whether it made sense for them in a clinical environment. However, the primary focus in part 3 was on the clinicians. Patient interviews were conducted via phone at the request of both patients. The interviews were recorded with consent and later transcribed.

The unique aspect lies in its exploration of text vignettes within clinical contexts, focusing on clinicians who interact with the patients described, and incorporating insights from their practical experience.

#### Analysis

Two interview guides were prepared, 1 for patient interviews and 1 for clinician interviews (Multimedia Appendix 1). These questions were grounded in the central inquiry: How can text vignettes generated by PRO data, as an alternative or supplement to traditional quantitative representations, improve dialogue and understanding between clinicians and patients regarding PROs?

All interviews were analyzed using qualitative content analysis using inductive coding to identify and describe patterns, categories, and meanings within the data [36]. The analysis was conducted using NVivo software (version 12.4.0; Lumivero) [37]. From the coding process, 4 main categories emerged: “recognition and confirmation,” “challenges with context and conceptual understanding,” “one size does not fit all,” and “reflections on using text vignettes.”

An example of a text vignette, as presented to both clinicians and patients, is shown in Textbox 2 (translated by the authors). This vignette is based on one of the patient’s responses to the READHY instrument.

**Text vignette based on Readiness and Enablement Index for Health Technology.**

She experiences having limited insight into her state of health, with limited knowledge of how to deal with health problems when they arise.

She finds that her health issues to some extent limit her from enjoying life.

She experiences having sufficient skills and techniques to deal with health problems as they arise and generally, experiences no emotional distress due to her health.

She feels adequately understood and supported by her social network and experiences adequate understanding and support from the healthcare professionals she has contact and access to.

She experiences having trouble understanding information about health and illness and may experience insufficient knowledge to have a conversation with others about this.

She finds that she has insufficient knowledge about how she can use technology to take care of her health and has insufficient knowledge of how to use technology to navigate healthcare systems.

She experiences having sufficient access to digital health systems that work and can be accessed by herself and others who need it, and that digital services can adequately adapt to her needs.

She finds that technology is helpful to a limited extent and helps her keep up with and take care of her health.

She feels comfortable with how her health data is used by others.

## Results

### Part 1

Four clusters with various levels of high, intermediate, and low average scores of scales within self-management, social capital, and digital health literacy were created (Table 3).

The mean value for each scale was presented for each of the 4 clusters that were informed. Values considered to represent a problematic level were indicated in red (<2.00), values considered to be below a sufficient level were indicated in yellow (2.00-2.50), and values considered to represent a sufficient level were represented in green (>2.50).

**Table 3:** Stratification using the Readiness and Enablement Index for Health Technology.

Metric (mean values)	Cluster 1 (n=16)	Cluster 2 (n=5)	Cluster 3 (n=11)	Cluster 4 (n=9)
heiQ3^a^: self-monitoring and insight	2.98	3.66	3.13	3.11
heiQ4: constructive attitudes and approaches	2.81	3.28	2.76	3.46
heiQ5: skill and technique acquisition	2.65	3.15	2.84	3.11
heiQ8: emotional distress	2.50	2.76	2.13	3.12
HLQ1^b^: feel understood and supported by health care providers	2.78	3.70	3.40	3.44
HLQ3: actively managing my health	2.68	3.40	2.92	2.68
HLQ4: social support for health	2.83	3.59	2.90	3.33
eHLQ1^c^: using technology to process health information	2.78	3.92	1.89	2.03
eHLQ2: understanding of health concepts and language	2.85	3.96	2.82	2.77
eHLQ3: ability to actively engage with digital services	2.95	3.96	1.99	2.65
eHLQ4: feel safe and in control	2.97	4.00	2.95	3.18
eHLQ5: motivated to engage with digital services	2.85	3.92	2.11	2.60
eHLQ6: access to digital services that work	2.86	3.96	2.42	2.97
eHLQ7: digital services that suit individual needs	2.78	3.80	2.04	2.68

^a^heiQ: Health Education Impact Questionnaire.

^b^HLQ: Health Literacy Questionnaire.

^c^eHLQ: eHealth Literacy Questionnaire.

On the basis of the thresholds, a focused text vignette was constructed almost as a narrative for each cluster together with key sociodemographic characteristics to create 4 text vignettes, wherein each represented an archetype of the profile—termed a “persona” (Multimedia Appendix 2). To simplify the information in this initial phase, only scales with high (initial green color) or low (initial red) average scores (>2.50 or <2.0) were included in the text vignettes, thereby creating focused text vignettes highlighting potential areas of barriers and resources.

EKW and LK then presented these text vignettes to colleagues in the Smart Inclusive Living Environments project. On the basis of the insights gained from the proof of concept with text vignettes at group or personal level, it was decided to proceed to part 2, focusing on the development of structured text strings that encompass high, intermediate, and low average score levels.

### Part 2

A total of 39 initial text strings were created, with 3 strings for each of the 13 READHY scales, reflecting different levels of knowledge, skills, or attitudes within each scale. These text strings were designed to align with the scale names, which summarize the relevant experiences. The initial text strings are included in Multimedia Appendix 3.

Immediate feedback from the first 3 participants indicated that they could easily recognize themselves in the text vignettes, found them meaningful, and saw how their responses were reflected in them.

In the first iteration of text vignette validation, participants largely recognized themselves but identified issues related to the structure of the text vignettes and the completion of the instrument. Feedback included suggestions to revise lengthy sentences and reduce academic language, with specific terms such as “health professionals” and “health-related concepts” being difficult to understand. Participants also struggled with the term “technology” used in the text vignette, such as in the description of scale eHLQ3.

On the basis of cognitive interviews, “healthcare providers” was changed to “healthcare professionals” and “health-related concepts” was simplified to “information of health and illness.” The order in which the scales were presented was also slightly changed. The order of HLQ4 and HLQ1 was adjusted to create a more natural flow when reading through the vignette. Similarly, the eHLQ items were reordered to enhance the coherence of the vignette.

In subsequent validation iterations, feedback was generally positive, with individuals finding the text vignettes understandable and recognizable. However, they struggled more with areas where they rated themselves as limited or insufficient. Some participants mentioned difficulties with the questionnaire due to the lack of a “don’t know” option and noted that terms such as “insufficient” felt too academic and unrepresentative of their self-view. The term “digital services” was also revised to “digital health services” to clarify its meaning. Participants suggested that discrepancies in text vignette descriptions might stem from specific episodes rather than their general experiences, and some participants requested more precise wording to better reflect their experiences. During iterations and refinement based on feedback, the understandability and recognizability of the vignettes improved, with individuals having an overall positive view of the text vignettes after final refinement.

The clinicians had a generally positive view about the generated READHY text vignettes. Feedback emphasized the need for clarity in highlighting that the text vignettes were based on a PRO instrument. The length of the text vignettes had mixed reviews. Feedback suggested categorizing content into taxonomic groups. The language was too academic, and adjustments had to be made to align with the instrument.

Clinicians see the value of using the text vignettes at the population level, allowing for generalization and description of specific populations based on competencies and characteristics. However, the current format may not be suitable for individual-level use, as it offers a simplified representation for personalized interaction. On the basis of input received from all participants, the strings were refined. The refined text strings are presented in Table 3 (translated from Danish to English by the authors). In the context of this study, we have used gender pronouns, but they should be adjusted to ensure relevance and significance within the context and setting.

### Part 3

There was a positive attitude among the clinicians and patients toward the text vignette format and this way of presenting and communicating PROs. The clinicians experienced being able to recognize patients in the text vignettes, and patients experienced being able to recognize themselves in their text vignettes. Although the text vignette format does not necessarily meet the specific needs, the textual presentation makes the information from PROs more accessible in an understandable way for both clinicians and patients.

There are different preferences for how dynamic the tool should be. Some prefer to select only specific dimensions, while others believe that the strength of the text vignettes comes from them providing the full picture insights and exposing possibly overlooked aspects. Clinicians who favor the text vignette suggest that color coding as a supplement to the text vignette would be helpful and that a schematic summary would help with readability, indicating that flexibility in the profile presentation is needed to accommodate different preferences. Some prefer text-based representations, while others find visual presentations more intuitive.

Clinicians may face language and comprehension challenges due to ambiguity in words and abstract concepts within the text vignettes. Ambiguity in words and concepts, such as “to some extent,” leaves clinicians uncertain about the severity of the patient’s problems. Abstract concepts such as “health” and “technology” also pose challenges in interpretation. The text vignettes highlight new angles in patients’ self-perception, health, motivation, and emotional distress, which clinicians find crucial.

Opinions differ on whether to highlight strengths or weaknesses. Some prefer insights into both aspects, while others prefer focusing solely on the weaknesses that are most likely to be approved. Clinicians acknowledge the value of using text vignettes at a population or group level for generalization but point out some limitations for individual-level use due to simplified representations. Emphasis on personal dialogue with patients remains crucial for addressing unresolved questions and determining personalized interactions.

From the patient’s viewpoint, there is self-recognition and understanding; however, engaging in discussions about text vignette representation proves challenging. It is essential to note that clinicians report based on their experience of interacting with patients, highlighting the dynamic nature of this interaction in understanding and interpreting the text vignettes. The clinician’s insights provide a valuable perspective for understanding the broader implications of text vignettes in a clinical setting.

## Discussion

### Principal Findings

This study presents a process for developing text vignettes based on PRO data, resulting in the creation of individual vignettes that incorporate all 13 scales of the READHY framework. The motivation for the development of these text vignettes was the challenges associated with presenting PRO data as numeric values or graphical representations, aiming to provide a clearer and more engaging way to present and communicate PRO information.

This findings of this study suggest an overall positive attitude toward the PRO data–informed text vignette format and this way of presenting and communicating PROs. Clinicians experienced being able to recognize patients in the text vignettes, and the patient’s experienced being able to recognize themselves in and understand the text vignettes describing themselves.

Considering the background emphasizing the challenges associated with graphical representations of PRO data, the findings suggest that PRO-informed text vignettes are understandable for clinicians and patients. While the text format may not perfectly align with all the needs and interests of clinicians and patients, it presents PRO data in a comprehendible manner, with the text vignettes presenting a recognizable description.

### Patients’ Perspective

Patients found elements of self-recognition in the text vignettes, although discussing their representation could be challenging. It is important to note that text vignettes also face challenges related to health literacy and differences in understanding, as highlighted in part 3. These challenges can pose significant barriers for individuals with low health literacy or those who cannot read. In such cases, regardless of the presentation format, additional support, such as reading the vignettes aloud, may be necessary. However, the written format generally makes the data more accessible and less complex.

Another potential challenge is that the threshold values used to select appropriate text strings may result in choices that are not recognized by the informants. For example, individuals scoring between 2.00 and 2.70 might not feel limited, or they may perceive issues in areas not fully captured by the items in a scale. This issue requires further exploration in future studies to determine whether scales can be used effectively or whether a focus on single items is more appropriate, particularly for those scoring at the extremes. This may be necessary as we work in the intersection of presenting data from psychometric valid scales reporting on latent variables and presenting numeric values as an interpretation of experiences. The patients’ experiences and feelings may sometimes be undermined or misinterpreted in this process.

### Clinicians’ Perspective

The data-informed text vignettes add a candid dimension to the information, making it directly engaging and allowing for the capture of new perspectives on patients that clinicians would not otherwise have focused on, although not all perspectives are equally relevant. It is especially the aspects of experienced support and patients’ own knowledge about health and illness that are important for clinicians to understand and gain perspective on. There are different preferences among clinicians regarding the format of the text vignettes; some prefer text-based representations, while others prefer visual presentations. Likewise, there are different preferences regarding how dynamic the text-based tool should be. Some experience a need to be able to select only specific dimensions, while others believe it is part of the strength of the vignettes that it presents the full picture and thus insight into possibly overlooked aspects. This indicates that there is a need for flexibility in the text vignette presentation to accommodate different preferences.

### Clinical Relevance

Clinicians using text vignettes stress the importance of technology that effectively addresses practical and treatment-specific needs. It remains imperative that the information within text vignettes aligns with the treatment being offered.

Clinicians emphasize that the central focus should be on understanding the condition and enabling patients to lead fulfilling lives. Reading the text vignettes prompts clinicians to reflect on aspects that may have been previously overlooked, serving as a reminder of the critical need for clinical relevance in any work involving PROs. It underlines that for PROs, and indeed text vignettes, to realize its full potential in creating value, it must align with the clinical context and be relevant to clinical practice.

### A More Straightforward Representation of PROs

In addressing some of the challenges with the quantitative representation of PROs, the use of text vignettes allows for a deeper understanding of PROs. Text vignettes recognize the complexities of patients’ experiences and provide a broader perspective. This approach prevents reducing patients to just numerical data, offering instead a more comprehensive view of an individual’s health. The text vignettes are presented in an understandable and consistent format, with transparency about how the collected information is applied. This transparency is crucial for patients’ effective use of PROs [38]. By transforming complicated numerical data into a clearer and more coherent text format, text vignettes offer a direct representation that ensures patients and clinicians can grasp details without relying on prior knowledge of graphical presentations. This textual format encourages conversation, minimizes ambiguity, and fosters dialogue for a better understanding of individual experiences.

### Promoting Dialogue for Mutual Understanding

A data-informed text vignette is not a universal solution, but rather an approach with both advantages and disadvantages that must be weighted and adapted to individual preferences and needs and that requires a more dynamic approach to meet needs. Recognizing these challenges suggests a need for meaningful conversations between patients and clinicians about aligning their understanding at a conceptual level. Such discussions offer a chance to clarify word meanings and ensure a common understanding of the patient’s experiences. This approach is essential for accurate and meaningful communication between patients and clinicians, ultimately improving the quality of care and treatment. Clinicians and patients may have different perspectives on what the patient reports in the PRO instrument. An example is how HLQ scores were not perceived in the same way by patients and their physicians, with a disconcordance ranging between 20% and 44% in the 9 HLQ scales [14]. Translating the data into text vignettes may ease the conversation and help identify these disconcordances for other PRO data also, ease the alignment of expectations, and provide a common understanding of the patient perspective.

### Bias Concern

Clinicians are generally receptive to text vignettes and the insights they provide. Many clinicians feel that the text vignettes offer a strong sense of the patients before any direct contact is made. However, clinicians also express concerns that the text vignette may make them biased in their encounter with the patient, which may affect their ability to understand and treat the patient objectively. It emphasizes the necessity of acquiring proficiency in tool use, particularly when presenting information in a text format. Being mindful of potential biases becomes central, especially in a context where the text vignette format might intensify the impact. This highlights the importance of a nuanced and skillful application of the tool, recognizing that biases are not exclusive to text vignette representation but should be carefully navigated, especially when conveyed in a confrontational textual manner.

### Strengths and Limitations

A strength of this study lies in the stepwise process of creating a dataset using a validated instrument, followed by data triangulation. This process is based on iteratively developed text lines that can be combined into cohesive text vignettes, forming complete paragraphs. The cocreation with individuals, people living with a chronic condition, municipalities, hospital-based clinicians, and 1 general practitioner helps ensure the validity and meaningfulness of the proposed solution.

The number of participants in each of the 3 parts may be considered a limitation. In part 1, convenience sampling was used among participants active in a telehealth service and was limited by availability. We do not find this to be a problem for the presented results, as we were able to create 4 profiles and personas that are meaningful and can serve to illustrate the principles used as background for parts 2 and 3. The number of participants in part 2 was determined through concurrent evaluation during data sampling. When the inclusion of additional informants no longer contributed new information, the sampling process was considered complete. By including data from 2 distinct sociodemographic areas, we aimed to capture a range of perspectives. On the basis of these arguments, we found the number to have sufficient information power for a proof-of-concept study [39]. This is also supported by most of the participants finding the text vignettes understandable and recognizable.

In particular, in part 3, the number of patients is likely to be too small, but we have included this part to illustrate how clinicians who are familiar with the patients respond to the text vignettes describing them. The findings need to be assessed in this light. Moreover, the results may differ from those that will be obtained using PRO data instruments that are developed for clinical purposes, such as European Organisation For Research And Treatment Of Cancer [40], The World Health Organization-Five Well-Being Index [41], or European Quality of life–5 Dimensions [42].

### Perspective

The study has used the READHY instrument to demonstrate how data-based text vignettes can help to understand patients and align expectations about what the data tell. During the field studies and in discussions afterward, we have received expressions of interest in setting up algorithms to convert other established PRO data results into text vignettes. This calls for abundant work in this field in collaboration with several PRO instrument owners and distributors, and we welcome collaboration in this field. It also calls for atomization programming interfaces that build on the proposed structures with filters based on cutoff values combined with generative artificial intelligence to refine the created text vignettes.

### Conclusions

The study demonstrates how text vignettes can be developed using PRO instruments, with individual scales as input strings. This provides an opportunity to present numeric values in a text format that is understandable and recognizable to most patients and clinicians. Some clinicians prefer text vignettes to other forms of presentations and find them especially useful in establishing a better understanding of a patient before initial contact. However, language and the arrangement of text vignettes can present challenges, underscoring the need for adaptation to specific needs and clinician training to ensure accurate interpretation of the text vignettes. Different preferences for text vignette formats highlight the need for a more dynamic and adaptable tool that can cater to individual needs and preferences.

## Appendix

Multimedia Appendix 1 Interview guides.

Multimedia Appendix 2 Group-based text vignettes.

Multimedia Appendix 3 Initial text strings based on scores.

## Abbreviations

COPD: chronic obstructive pulmonary disease
eHLQ: eHealth Literacy Questionnaire
heiQ: Health Education Impact Questionnaire
HLQ: Health Literacy Questionnaire
OPHELIA: Optimizing Health Literacy and Access
PRO: patient-reported outcome
READHY: Readiness and Enablement Index for Health Technology
