# Supplementary material for: Creation of Text Vignettes Based on Patient-Reported Data to Facilitate a Better Understanding of the Patient Perspective: Design Study
Source: JMIR Hum Factors. 2025 Feb 5;12:e58077. doi: 10.2196/58077 (PMC11840378; doi:10.2196/58077)
Supplement: Multimedia Appendix 1 [file humanfactors_v12i1e58077_app1.docx]

### Multimedia Appendix 1 – Interview guides

*The interview guides have been translated from Danish to English by the authors.*

##### **Interview guide for clinicians**

*Introduction*

Short introduction of the researcher, and to the purpose of the interview.

Brief introduction of the informant – who are they?

*Purpose of the interview*

To uncover whether the vignettes make sense to the clinicians, if they can facilitate a conversation about PRO and the individual’s need for support, what may be missing from the text vignettes, or whether the vignettes do not make sense at all.

**Questions**

1. Are you used to using Patient-Reported Information (PRO) in your work? How is it presented?
2. Do you have any method to uncover technology readiness among patients?
3. Do you see a need to be able to talk to citizens about information presented in the vignette?
4. How do you typically structure the conversation about PRO?
5. What are your thoughts about data from the questionnaire being presented in a text format?
6. What are your immediate experiences/thoughts about the vignette?
7. Do you find the vignettes readable and understandable? Is it too long? Too much text? Too little text?
8. Do you think the vignettes can provide a different starting point for having a conversation?
9. Do you experience the vignettes as a help to better understand the citizen challenges and strengths? why/why not? Do you recognize the citizen in the vignette?
10. Do you experience the vignettes as a help to introduce technology to the citizen? why/why not?
11. Do you think the vignettes can make the conversation about using the technology easier?
12. What do you think about having PRO presented this way?
13. Do you think you can use the vignettes to have a better conversation about technology and citizens’ need for support?
14. What barriers do you see to the application of text vignettes?
15. What benefits do you see to the application of text vignettes?
16. Can you imagine applying the vignettes in practice? why/why not
17. Do you think there is something particularly important to highlight in the vignettes?
18. Do you think something is missing from the vignettes?
19. Do you think there is something in the vignettes that makes no sense at all?
20. Do you have anything to add or any questions?

##### Interview guide for patients

*Introduction*

Short introduction of the researcher, and to the purpose of the interview.

Brief introduction of the informant – who are they?

*Purpose of the interview*

To uncover whether the vignettes make sense to the clinicians, if they can facilitate a conversation about PRO and the individual’s need for support, what may be missing from the vignettes, or whether the vignettes do not make sense at all.

**Questions**

1. How did you experience reading the vignettes?
2. Did you find them difficult to understand?

- Did you find them long? Too much text? Too little text?

1. Do you find that you could recognize yourself in the vignettes?
2. Do you feel represented in it?
3. Have you filled out questionnaires in connection with treatment before? If yes: Have you received feedback on them? How?
4. Do you think you understand what the questionnaire is aimed at when you see the vignettes? Is it a different understanding than the one you had before?
5. Do you perceive yourself in a different way than what is presented in the vignettes?
6. Do you recognize your own answers, from the questionnaire in this text?
7. Do you become aware of anything about yourself that you would like to talk to the nurses about when you read the text?
8. Is there anything in the text you think should be elaborated?
9. Would it be beneficial for you to have this text with you when having a conversation with a nurse? why/why not?
10. If you were to have a chat with the nurses about what you are good at and what you have difficulty with, would this text help you describe that?
11. Do you find that you need to be able to talk to nurses about technology?
12. Is there anything in this text that you particularly feel the need to be able to talk to a nurse about?
13. Is there anything in this vignette that you explicitly don't feel the need to talk to a nurse about?
14. Do you have anything to add or any questions?
