# Supplementary figures and images for: Creation of Text Vignettes Based on Patient-Reported Data to Facilitate a Better Understanding of the Patient Perspective: Design Study
Source: JMIR Hum Factors. 2025 Feb 5;12:e58077. doi: 10.2196/58077 (PMC11840378; doi:10.2196/58077)

### Multimedia appendix 2. Group based text vignettes


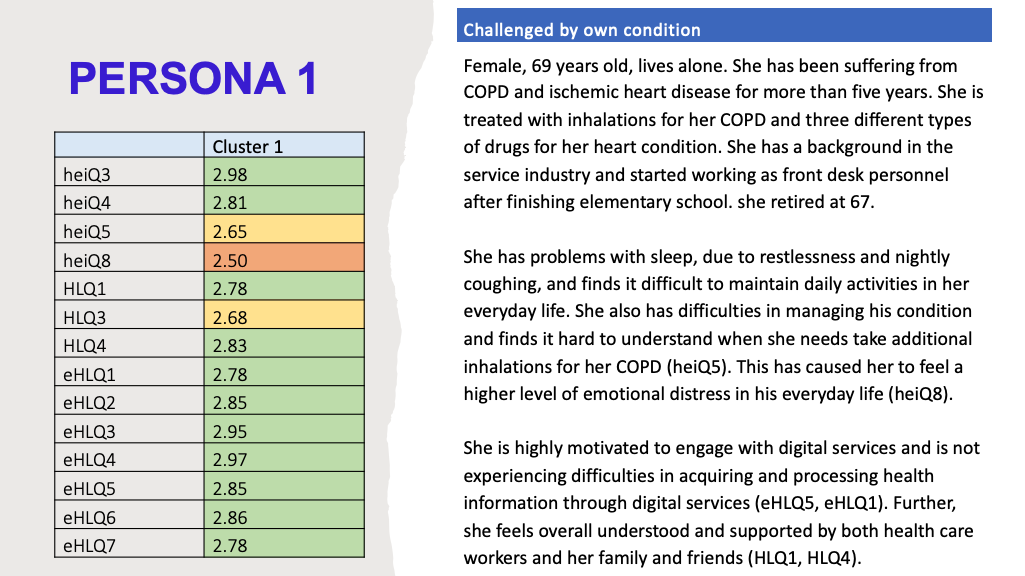


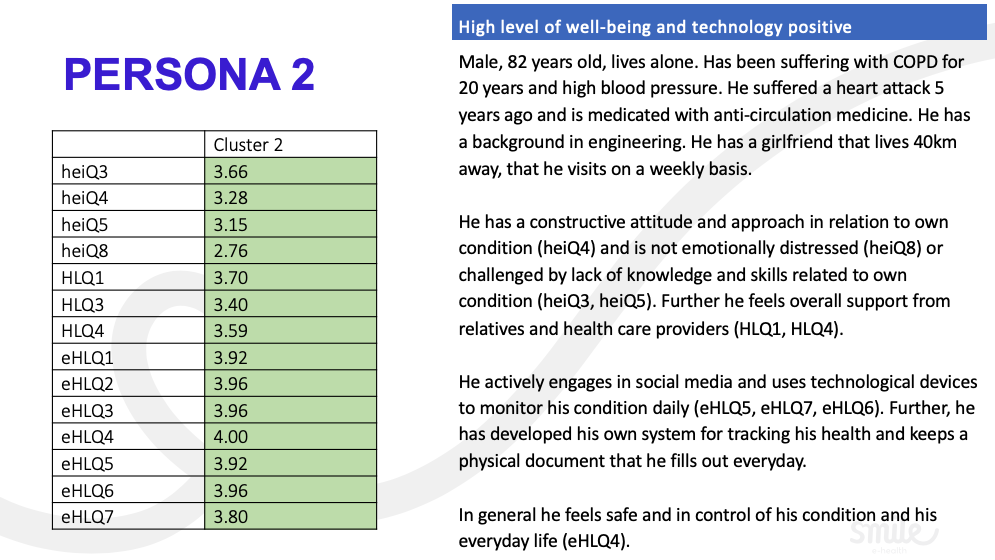


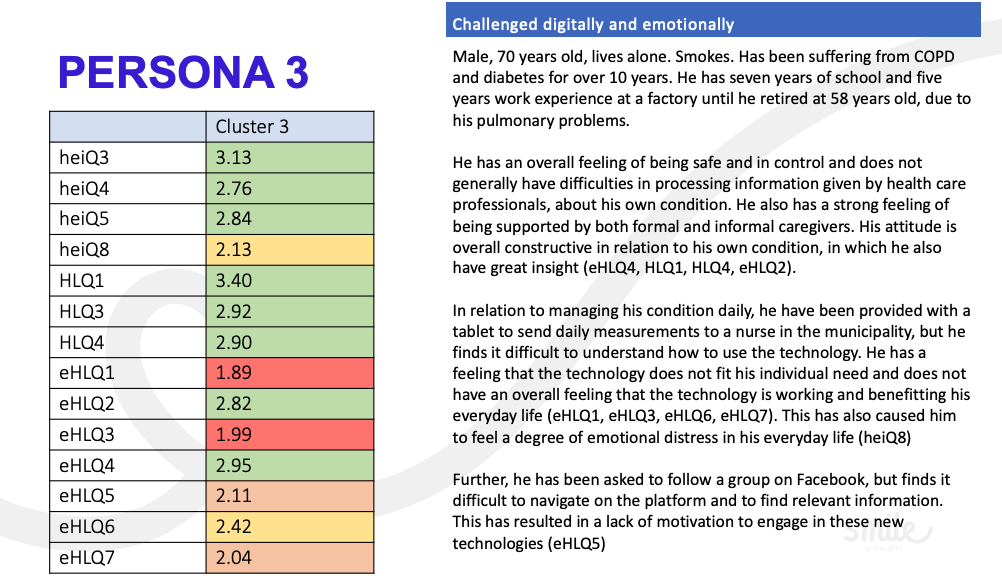


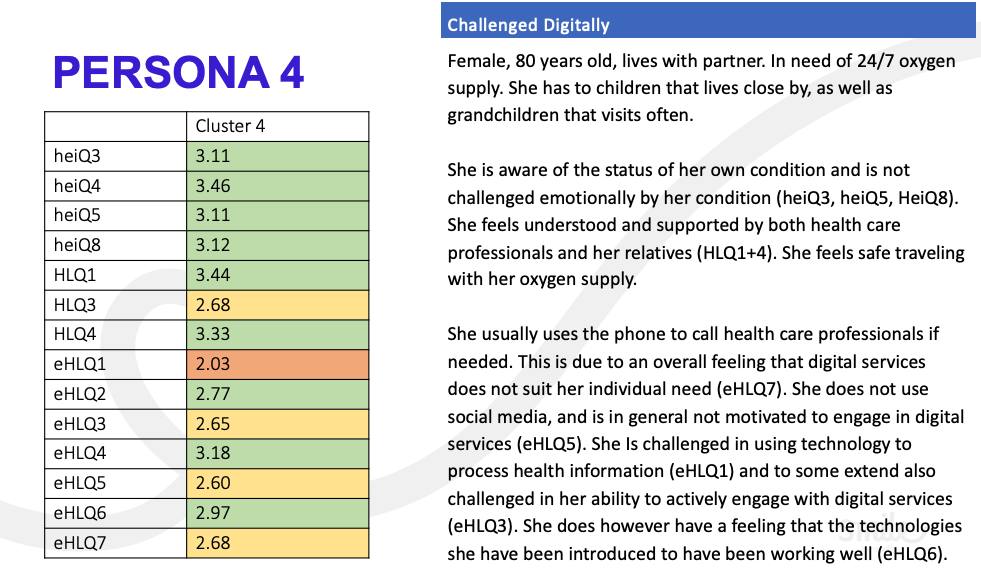

Supplement: Multimedia Appendix 2 [file humanfactors_v12i1e58077_app2.docx]
