# Supplementary material for: Creation of Text Vignettes Based on Patient-Reported Data to Facilitate a Better Understanding of the Patient Perspective: Design Study
Source: JMIR Hum Factors. 2025 Feb 5;12:e58077. doi: 10.2196/58077 (PMC11840378; doi:10.2196/58077)
Supplement: Multimedia Appendix 3 [file humanfactors_v12i1e58077_app3.pdf]

Multimedia Appendix 3. Initial text strings based on scores.

| Scale/Description | Under 2.00 | Over 2.00 to 2.70 | Over 2.70 |
|---|---|---|---|
| HeiQ3: Self-monitoring and insight | Has doubtful and insufficient insight into and control over their own health condition. | Has limited insight into and control over their own health condition. | Has good insight into and control over their own health condition. |
| HeiQ4: Constructive attitudes and approaches | and has a non-constructive attitude and approach towards it. | and has a somewhat constructive attitude and approach towards it. | and has a constructive attitude and approach towards it. |
| HeiQ5: Skill and technique acquisition | Lacks skills and techniques to handle health problems when they arise. | Has inadequate skills and techniques to handle health problems when they arise. | Has sufficient skills and techniques to handle health problems when they arise. |
| HeiQ8: Emotional distress | (and) experiences a high degree of emotional stress due to their health. | (and) experiences a slight emotional stress due to their health. | (and) experiences no emotional stress due to their health. |
| HLQ1: Feel understood and supported by healthcare providers | Feels misunderstood or unsupported by their social network. | Feels only partially understood and supported by their social network. | Feels well and adequately understood and supported by their social network. |
| HLQ4: Social support for health | (but) experiences a lack of understanding and support from the healthcare providers they have contact with. | (but) does not experience sufficient understanding and support from the healthcare providers they have contact with. | (and) experiences good and adequate understanding and support from the healthcare providers they have contact with. |
| eHLQ2: Understanding of health concepts and language | Lacks understanding of health-related concepts and the health-related language and has limited knowledge of their own health condition. | Has limited understanding of health-related concepts and the health-related language and has limited knowledge of their own health condition. | Has a good understanding of health-related concepts and the health-related language, with good knowledge of their own health condition. |
| eHLQ1: Using technology to process health information | Has insufficient knowledge of how to use technology to | Has limited knowledge of how to use | Has sufficient knowledge of how to use technology to |

| | | | |
|---|---|---|---|
| | manage their own health. | technology to manage their own health. | manage their own health. |
| eHLQ3: Ability to actively engage with digital services | Has poor knowledge of how to use technology to benefit themselves, interact with systems, and find health information relevant to themselves. | Has limited knowledge of how to use technology to benefit themselves, interact with systems, and find health information relevant to themselves. | Has good knowledge of how to use technology to benefit themselves, interact with systems, and find health information relevant to themselves. |
| eHLQ4: Feel safe and in control | Feels a lack of control over their data and lacks trust in how their data is handled and used by healthcare providers. | Feels limited control over their data and lacks trust in how their data is handled and used by healthcare providers. | Feels they have control over their data and trust how their data is handled and used by healthcare providers. |
| eHLQ6: Access to digital services that work | Has insufficient access to digital health services that work and can be accessed by herself and others who need them. | Has limited access to digital health services that work and can be accessed by herself and others who need them. | Has sufficient access to digital health services that work and can be accessed by herself and others who need them. |
| eHLQ7: Digital services that suit individual needs | and she feels that the digital services do not adequately adapt to her needs. | and she feels that the digital services only partially adapt to her needs. | and she feels that the digital services adequately adapt to her needs. |
| eHLQ5: Motivated to engage with digital services | (and) is not motivated to engage with these services. | (and) is not motivated to engage with these services. | (and) is not motivated to engage with these services. |
